# Giant Fibrous Dysplasia of the Sixth Rib Managed in a Rural, Resource‐Limited Setting: A Case Report

**DOI:** 10.1155/cro/3789997

**Published:** 2026-09-01

**Authors:** Sagarika Bhole, T. R. V. Wilkinson, Sushant Nair, Ashish Tiwari

**Affiliations:** ^1^ Department of General Surgery, N.K.P. Salve Institute of Medical Sciences, Nagpur, India; ^2^ Associate Professor, Department of General Surgery, N.K.P. Salve Institute of Medical Sciences, Nagpur, India

**Keywords:** case report, chest wall reconstruction, fibrous dysplasia, latissimus dorsi flap, monostotic fibrous dysplasia, rib tumor

## Abstract

Fibrous dysplasia (FD) is a benign bone disorder characterized by replacement of normal bone with fibrous tissue and immature woven bone. Although rib involvement is not uncommon, large symptomatic lesions are rare. We report a case of giant monostotic FD of the sixth rib managed successfully in a resource‐limited rural setting. A 22‐year‐old man presented with a progressively enlarging, painful swelling over the left chest wall for 3 years. Examination revealed a hard, immobile mass measuring 10 × 6 cm. Imaging showed a well‐defined expansile lytic lesion with ground‐glass matrix arising from the left sixth rib without cortical breach. Core biopsy confirmed FD. Owing to the lesion size, symptoms, and limited access to thoracoscopic facilities, open posterolateral thoracotomy with en bloc rib resection was performed. The resulting 8 × 6 cm defect was reconstructed using rib approximation without prosthetic mesh and covered with a pedicled latissimus dorsi flap. The postoperative course was uneventful, with complete recovery and no recurrence at 1‐year follow‐up. This case highlights that complete surgical excision provides excellent clinical outcomes for symptomatic rib FD and demonstrates that effective, low‐cost reconstruction without synthetic materials is feasible in selected patients in resource‐constrained settings.

## 1. Introduction

Fibrous dysplasia (FD) is an innocent condition of the skeleton in which normal bone is substituted by fibrous tissue and immature woven bone, leading to structural weakness and susceptibility to fractures [1–3]. The condition is categorized into two types according to the skeletal site affected: monostotic FD, where one bone is involved, and polyostotic FD, where more than one bone site is involved. Approximately 30% of all benign tumors of the chest wall bones are rib tumors, which occur four times more than their polyostotic FD counterpart [4]. Even though this disorder can occur in any bone site in the body, some of the commonest sites are the long bones, facial bones, and ribs. Most of the cases are detected incidentally on radiological examination, but larger cases might present with symptoms such as pain, deformity, fractures, or malignancy.

Postzygotic activating mutations of the GNAS gene are the cause of pathogenesis FD, which cause constitutive activation of the cyclic adenosine monophosphate (cAMP) signaling pathway. This pathological signaling interferes with normal osteoblastic differentiation and bone remodeling leading to the replacement of mature lamellar bone by fibro‐osseous tissue of immature woven bone in a fibrous stroma [5]. Radiologically, FD usually presents as an expansile intramedullary lesion with a distinctive matrix of ground glass, but the imaging appearance can be similar to other benign bone tumors. This means that histopathological confirmation usually needs to be conducted in order to arrive at a definite diagnosis.

The treatment of rib FD is guided by the patients′ symptoms, tumor size, anatomic position, and the possibility of complications. In cases where there are no symptoms, observation is the treatment strategy, but in cases where the tumor is symptomatic, enlarging, causing cosmetic deformity, is of uncertain diagnosis, or of possible malignant degeneration, then surgery becomes necessary. The need for the degree of resection influences surgical technique and chest wall reconstruction as well as the stability of the thorax after surgery.

The case report, written as per the CARE (Case Report) guidelines, presents an abnormally large (10 × 8 cm) monostotic FD of the left sixth rib which was successfully treated by open thoracotomy with en bloc resection, chest wall repair with rib approximation, and a pedicled latissimus dorsi muscle flap in the absence of synthetic mesh and rigid plating. The case presents a viable surgical process of treating giant rib FD in a well‐chosen patient and shows that good clinical and functional results are possible with the use of autologous reconstruction even in the context when state‐of‐the‐art reconstructive tools are not easily accessible.

## 2. Case Presentation

### 2.1. Patient History

The patient was a 22‐year‐old male who visited the Surgery Outpatient Department with a chief complaint of swelling on the left side of his chest for 3 years. Swelling in the initial stage was asymptomatic and insidious, with a small size of about 3 × 3 cm. There was a significant growth in the size of swelling during the past 6 months that made the patient come to hospital for further management. The swelling initially was painless; however, pain started 6 months before presenting to the hospital. The pain was dull, aching, and localized in the swelling but without any radiation.

Patient did not have any notable medical or surgical history and had not been hospitalized before. He had no known drug or food allergies or regular medications given to him. The family history of bone disorders, skeletal dysplasia, or malignancy was nonexistent. He had a poor social history. Other than the gradually increasing swelling of the chest wall and pain, he did not report any history of trauma, fever, weight loss, respiratory problems, and other constitutional complaints. Table 1 presents a chronological summary of the presentation, diagnostic work up, surgical management, and follow up of the patient.

**Table 1:** Patient clinical timeline.

Timeline	Clinical event
Approximately 3 years before presentation	Left chest wall swelling first noticed (approximately 3 × 3 cm), initially painless.
Approximately 6 months before presentation	Rapid enlargement of the swelling with onset of intermittent dull aching pain.
May 2024	Presentation to the Surgery Outpatient Department.
May 2024	Clinical examination, chest X‐ray, contrast‐enhanced CT, and CT‐guided core biopsy performed, confirming fibrous dysplasia.
May 17, 2024	Open posterolateral thoracotomy with en bloc sixth rib resection and chest wall reconstruction.
Postoperative Day 4	Intercostal drain removed.
Postoperative Day 10	Sutures removed.
June 2024 (30‐day follow‐up)	Complete recovery with stable chest wall and no evidence of recurrence.
June 2025 (1‐year follow‐up)	No clinical or radiological evidence of recurrence.

### 2.2. Clinical Examination and Diagnostic Assessment

**Figure 1:**
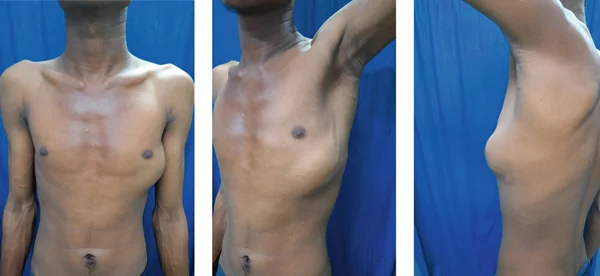
Clinical images showing chest wall swelling in anterior, anterolateral, and lateral views.

Physical examination revealed an isolated tumor mass that was 10 × 6 cm in size and present in the lateral chest wall over the left anterior axillary line. The lesion was firm to touch and fixed to the underlying bone, without any overlying skin fixation. There was no presence of skin changes, ulceration, or local inflammation. The clinical picture of the tumor from different angles is shown in Figure 1.

The prominent swelling in the lateral aspect of the chest wall is clearly evident in Figure 1, with unbroken skin without any signs of inflammation. This clinical presentation indicated that the swelling might be originating from the underlying chest wall, requiring further imaging studies.

**Figure 2:**
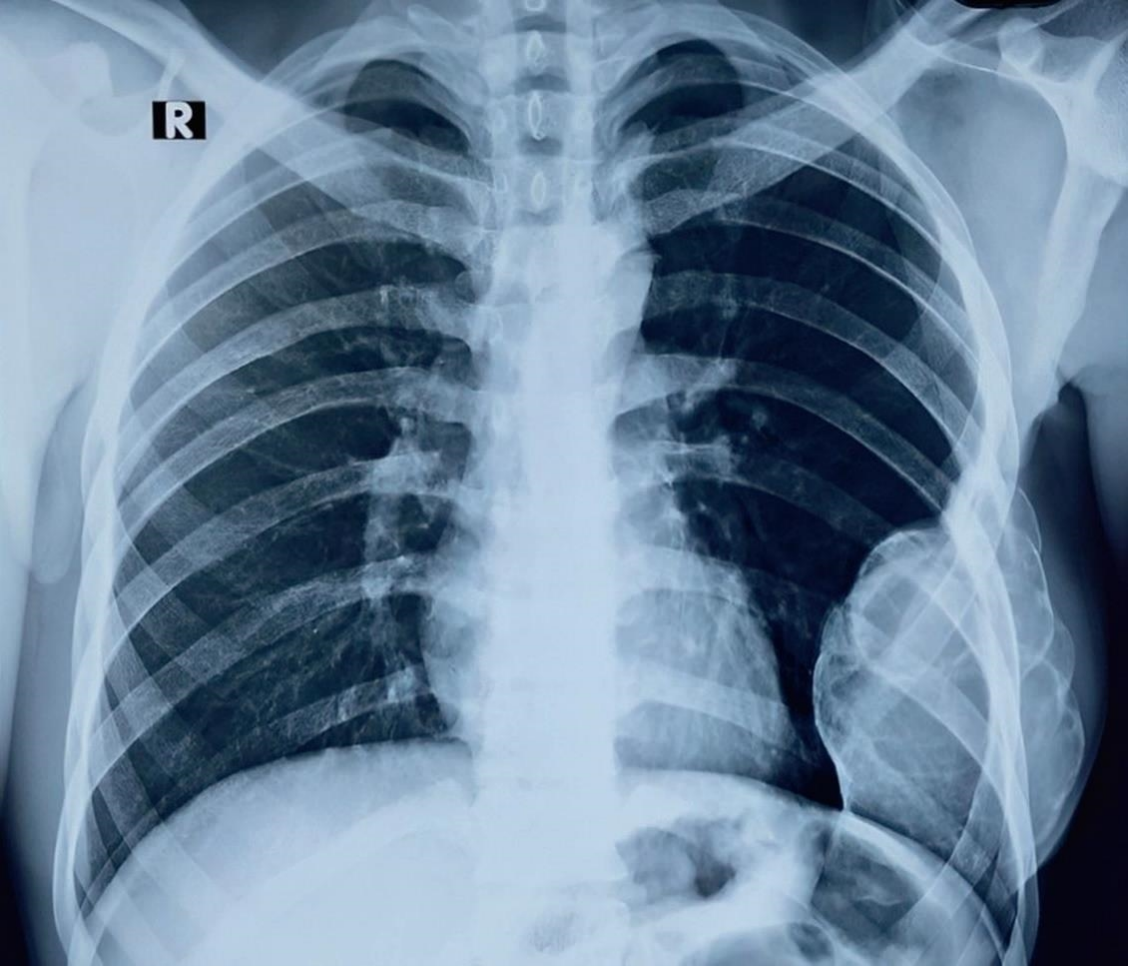
Chest X‐ray showing a well‐defined, expansile lytic lesion involving the sixth rib.

Initial radiological assessment was performed using a chest radiograph, which, as seen in Figure 2, revealed a distinct expansile lytic lesion affecting the left sixth rib.

An expansile lytic lesion affecting the left sixth rib is seen in Figure 2, supporting the presence of a benign osseous chest wall lesion and indicating the need for further characterization with cross‐sectional imaging.

**Figure 3:**
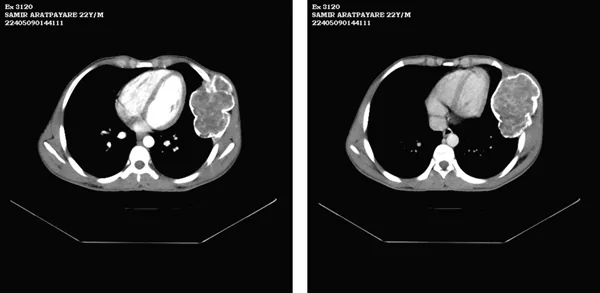
Contrast‐enhanced chest CT cross‐section showing the tumor.

A single, massive, well‐defined, multilobulated, intramedullary, expansile lytic rib lesion spanning roughly 9.6 × 7 × 6.5 cm (120 HU) emerged from the anterolateral part of the left sixth rib on a contrast‐enhanced computed tomography (CT) scan of the chest in May 2024. Medullary expansion was noted with no evidence of cortical breach or extraosseous extension. A heterogeneous ground‐glass matrix was present without chondroid or osteoid calcifications. No hemorrhage or dominant cystic spaces were identified. On postcontrast study, no enhancement was observed. The lesion demonstrated preserved fat planes with the surrounding structures. These radiological findings were suggestive of a benign neoplastic lesion originating in the left sixth rib. The differential diagnoses included aneurysmal bone cyst, osteoblastoma, chondroblastoma, and FD of the rib. The characteristic CT findings are illustrated in Figure 3.

Figure 3 demonstrates a large expansile intramedullary lesion arising from the left sixth rib with a characteristic heterogeneous ground‐glass matrix, a preserved cortex, and an absence of extraosseous extension, findings that favor a benign fibro‐osseous lesion.

To establish a definitive diagnosis, a CT‐guided core biopsy of the lesion was performed. Histopathological examination demonstrated irregular woven bone trabeculae embedded within a fibrous stroma, confirming the diagnosis of FD. Following histopathological confirmation and multidisciplinary surgical evaluation, definitive operative management was planned.

### 2.3. Surgical Management

Following discussion with the surgical team, surgical resection of the lesion was planned because of the patient′s pain, progressive increase in lesion size, and chest wall deformity. An open left posterolateral thoracotomy was scheduled because of the inaccessibility of thoracoscopic equipment in our rural hospital, the unwillingness of the patient to have surgery in a different hospital, and financial issues. Patient′s informed consent was obtained.

The patient was placed on airway protection under general anesthesia in the right lateral decubitus position on May 17, 2024, to have left posterolateral thoracotomy. A standard cleaning and draping was followed with the incision over the swelling being elliptical. By dissection through the skin, subcutaneous tissue, and muscle, a 10 × 8 cm expansile FD mass originating in the sixth rib, without invasions into the soft tissue, pleural adhesions, or vascularity, was identified. The mass was carefully removed together with the structures around it, followed by resection of the sixth rib as a block with a 2 cm bony margin; the medial incision was made 1 cm lateral to the sternal border, and the lateral incision was at the midaxillary line by means of a Gigli saw. The specimen was sent for histopathological examination.

The resulting 8 × 6 cm chest wall defect was reconstructed by approximating the fifth and seventh ribs using Prolene 1‐0 round body sutures, which provided satisfactory chest wall stability following single‐rib resection. A pedicled latissimus dorsi muscle flap was subsequently harvested and rotated anteriorly to provide soft tissue coverage and obliterate the postoperative dead space.

The latissimus dorsi muscle flap was selected because it provides a large, reliable regional flap with a constant thoracodorsal vascular pedicle, an excellent arc of rotation, and minimal donor‐site morbidity. The flap was elevated while carefully preserving the thoracodorsal vascular pedicle and was rotated anteriorly to completely cover the chest wall defect. It was then securely inset over the reconstructed chest wall to reinforce soft tissue coverage, protect the pleural cavity, and effectively obliterate the dead space. As adequate chest wall stability was achieved by approximation of the adjacent ribs after resection of a single rib, additional prosthetic mesh or rigid plating was not considered necessary.

**Figure 4:**
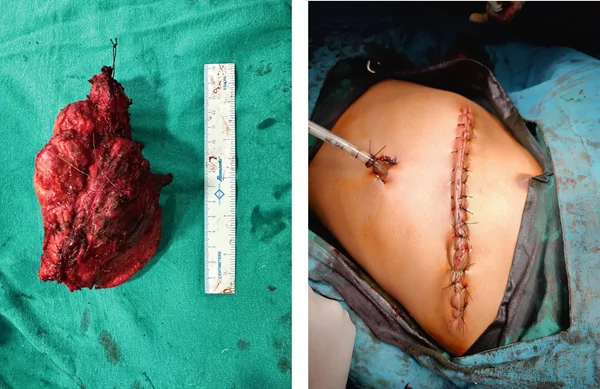
Intraoperative image showing excised specimen and postoperative image just after surgery showing intercostal drain.

Hemostasis was secured, with an estimated blood loss of 400 mL. An intercostal drain was placed, fixed, and connected to an underwater seal drainage system. Vicryl sutures Size 2‐0 round body were used to close the muscle and subcutaneous planes, whereas Ethilon sutures Size 3‐0 reverse cutting were used for the closure of the skin, and dressings were placed in accordance with standard procedures. The duration of surgery was 200 min, with no intraoperative complications noted. The major intraoperative observations and the postoperative presentation are illustrated in Figure 4.

The successful total excision of the sixth rib lesion and the immediate postoperative reconstruction are demonstrated in Figure 4. Rib approximation was used to obtain the stability of the chest wall, whereas the pedicled latissimus dorsi muscle flap was used to ensure long‐term coverage of soft tissues without the use of a prosthetic net or rigid fixation.

### 2.4. Postoperative Course and Follow‐Up

**Figure 5:**
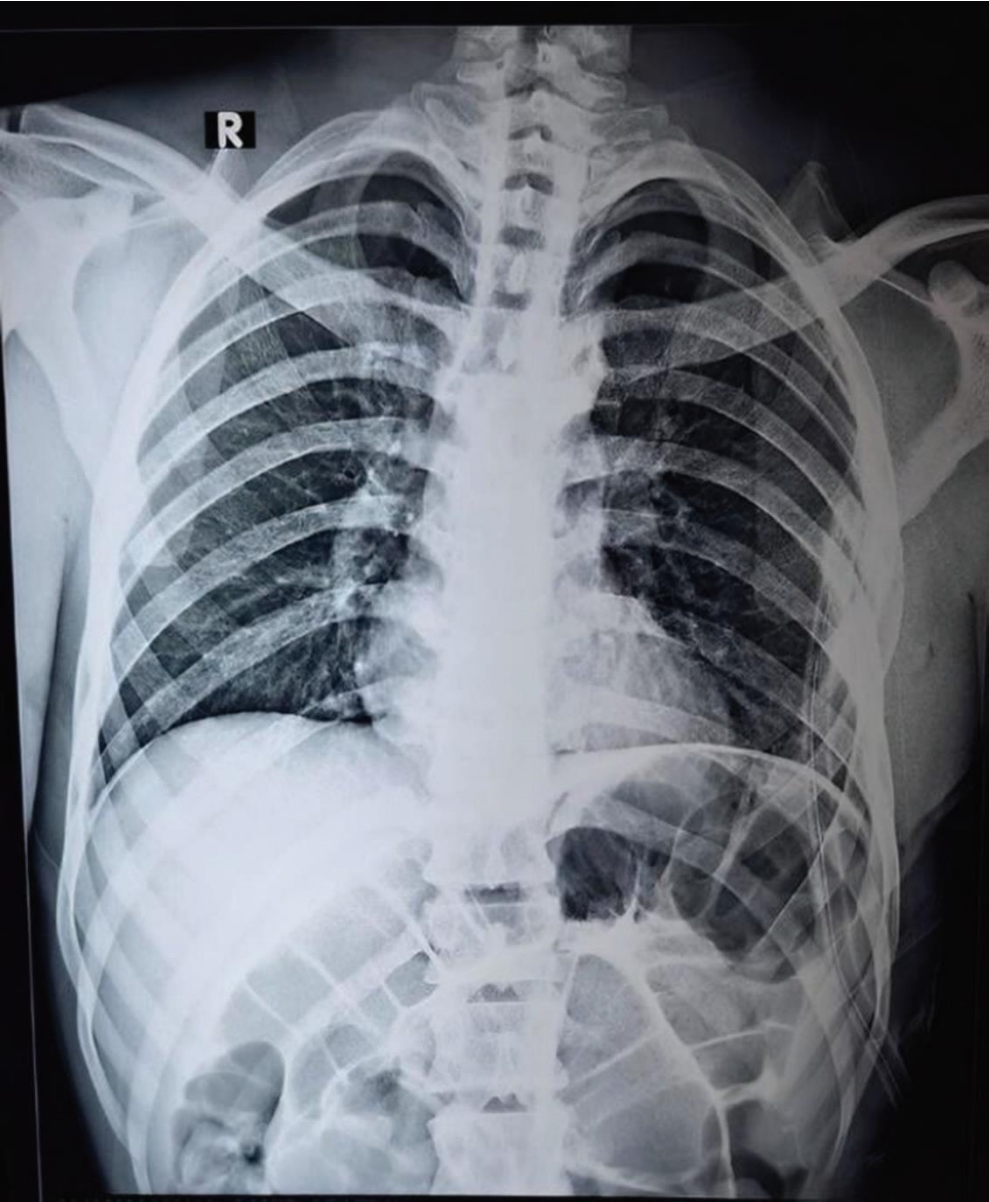
Postoperative chest X‐ray showing the intercostal drain in situ.

Postoperative recovery was normal. Intercostal drain removal on Day 4 and suture removal on Day 10 of postoperative recovery resulted in the patient recovering well. The patient was also hemodynamically stable and did not have respiratory compromise during the postoperative period. Close monitoring of the viability of the pedicled latissimus dorsi muscle flap was also followed, it was satisfactory during the recovery period, and there was no report of flap ischemia, flap necrosis, wound infection, formation of seroma, and wound dehiscence. An immediate postoperative chest radiograph after surgery showed that the lung expansion was satisfactory with intercostal drain in place, as illustrated in Figure 5.

Figure 5 displays good lung expansion after surgery and good placement of intercostal drain after a chest wall reconstruction without pneumothorax and other acute postoperative issues.

**Figure 6:**
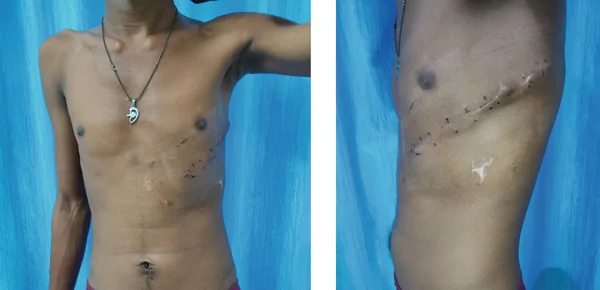
Postoperative image showing a well‐healed surgical scar over the left chest wall following suture removal.

After the sutures were removed, the surgical wound healed satisfactorily with good cosmetic results and no signs of wound‐related complications. The patient had good shoulder functioning and no restrictions in the upper limb motions, which means that the latissimus dorsi muscle flap maintained its functional integrity. Figure 6 depicts the postoperative scar that is well‐healed.

Figure 6 shows that the chest wall reconstruction with the pedicled latissimus dorsi muscle flap has satisfactory wound healing with acceptable cosmetic result.

**Figure 7:**
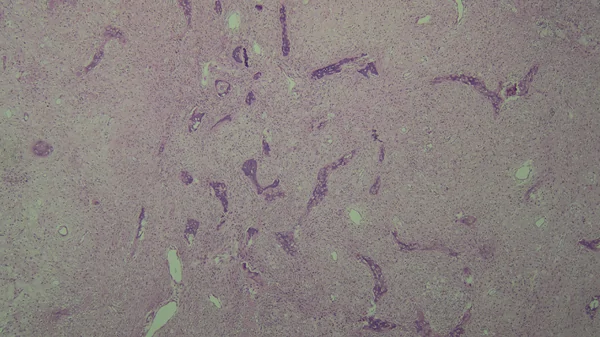
Histopathology image (10x) showing irregular woven bone trabeculae without osteoblastic rimming, embedded in fibrous stroma with spindle cells.

**Figure 8:**
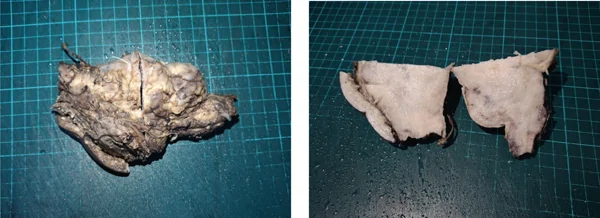
Gross specimen of the resected tumor and cut section of the tumor.

A well‐circumscribed lesion of uneven, branching, and anastomosing trabeculae of woven bone with typical C‐ and S‐shaped morphology without osteoblastic rimming was shown by the histology of the removed material. These bony trabeculae were embedded within a fibrous stroma containing cytologically bland spindle cells with plump ovoid nuclei and inconspicuous nucleoli. No mitotic figures were observed. However, the histopathologic findings were indicative of FD. No special staining techniques or immunohistochemistry had to be used, and there was no tumor at the surgical margins. Microscopic appearances are illustrated in Figure 7, whereas gross appearance of the excised specimen is illustrated in Figure 8.

The diagnosis is confirmed by Figure 7, which shows the distinctive histological characteristics of FD, such as irregularly woven bone trabeculae without osteoblastic rimming within a bland fibrous stroma.

The gross appearance of the lesion of the excised sixth rib is shown in Figure 8, demonstrating the expansile character of the tumor and being consistent with preoperative imaging.

During the 30‐day follow up performed in June 2024, the patient was asymptomatic with excellent recovery. The wound healed, and the postoperative CT showed complete expansion of the lung with a normal chest wall outline and without any signs of tumor recurrence. There was no paradoxical chest wall movement, respiratory compromise, or lung herniation, confirming satisfactory chest wall stability following reconstruction.

At the 1‐year follow‐up in June 2025, the patient remained asymptomatic with no clinical or radiological evidence of recurrence. The reconstructed chest wall maintained its stability, the latissimus dorsi flap remained healthy and incorporated, and normal daily activities had been resumed without functional limitation. Complete pain relief and satisfaction with the cosmetic and functional outcomes were reported, with no respiratory discomfort during routine activities. The overall postoperative outcome demonstrated excellent functional recovery following en bloc rib resection and autologous chest wall reconstruction.

## 3. Discussion

Primary chest wall tumors are uncommon, with benign lesions including osteochondroma, FD, chondroma, and eosinophilic granuloma [6]. Monostotic FD of the rib is a rare benign fibro‐osseous lesion that is often detected incidentally; however, larger lesions may present with pain, progressive deformity, pathological fractures, or, rarely, malignant transformation [5, 7]. The present case is noteworthy because of the giant size of the sixth rib lesion and its successful management with complete surgical excision and autologous chest wall reconstruction.

Management of FD depends on symptoms, lesion size, anatomical location, and the risk of complications. These small lesions are usually dealt with in a conservative way through clinical and radiological monitoring. Bisphosphonates have proven to be effective for treating painful polyostotic FD, reducing the bone pain; however, there is no evidence for the use of bisphosphonates in giant monostotic rib lesions [8]. In our case, pain progression, increase in the lesion size, cosmetic deformity, and pathological biopsy results made surgery the best solution in comparison with observation and pharmacotherapy.

Full surgical excision is still the treatment of choice in symptomatic rib FD and offers conclusive histopathological assessment to rule out the unlikely possibility of malignant transformation, and in larger defects, prosthetic replacement [9]. Despite increasingly reported cases with thoracoscopic rib resection because of its reduced invasiveness, published cases often depend on special instruments and, in the largest cases, prosthetic replacement. Our patient, on the contrary, was effectively treated with open posterolateral thoracotomy and total en bloc resection followed by autologous chest wall reconstruction, giving excellent exposure, total tumor resection, and good functional and cosmetic results.

It is also essential to reconstruct the chest wall after its resection. Traditionally, prosthetic mesh, titanium plates, methyl methacrylate, or biological materials are used to re‐establish thoracic stability with defects greater than 5 cm. Although these techniques offer great structural support, they lead to higher costs of operation and could be related to complications involving implants. In the current case, the chest wall was stabilized by bringing the adjacent ribs together when the single rib was resected with the pedicled latissimus dorsi muscle flap, offering long‐term vascularized soft tissue cover and effective dead‐space obliteration. The lack of thoracoscopic instrumentation and the financial limitations of the patient also played a role in the selection of this approach to reconstruction, proving that personalized surgical planning with the use of established resources can have outstanding results without endangering patient safety.

The main strength of this report is the effective treatment of a giant symptomatic sixth rib FD with full surgical resection and autologous reconstruction without any prosthetic materials. Nevertheless, the results are confined because of the single‐patient design and 1‐year follow‐up that limit the generalization and assessment of the stability of the chest wall over time. Nevertheless, this case emphasizes rib approximation and a pedicled latissimus dorsi muscle flap as a viable, replicable, and affordable choice in reconstructing carefully selected patients, especially when resources are constrained. Subsequent research using larger cohorts and extended follow‐up is required to confirm the functional outcomes over the long term and establish the best indicators to use for autologous or prosthetic chest wall reconstruction.

## 4. Conclusion

In symptomatic monostotic FD of the rib, en bloc excision of the involved segment provides effective symptom relief and enables definitive histopathological evaluation to exclude the rare possibility of malignant transformation. Early diagnosis through core biopsy and appropriate surgical management are associated with excellent clinical outcomes in patients with large symptomatic lesions. This case highlights the importance of individualized surgical planning and demonstrates that autologous chest wall reconstruction can be a feasible and effective option in carefully selected patients.

## Author Contributions

Designed research: S.B., T.R.V.W., S.N., and A.T. Performed research: S.B. and T.R.V.W. Analyzed data: S.B. and T.R.V.W. Data acquisition: S.B., T.R.V.W., S.N., and A.T. Writing original draft: S.B and T.R.V.W. Review and editing: S.B., T.R.V.W., S.N., and A.T.

## Funding

No funding was received for this manuscript.

## Disclosure

All authors have read and agreed to the published version of the manuscript.

## Ethics Statement

Written informed consent for publication of clinical details and images was obtained from the patient. As this is a single case report without prospective research intervention, formal ethics committee approval was not required per institutional guidelines. The study conforms to the provisions of the Declaration of Helsinki.

## Conflicts of Interest

The authors declare no conflicts of interest.

## Supporting information


**Supporting Information** Additional supporting information can be found online in the Supporting Information section. Supporting Information. File S1: Completed CARE checklist for the present case report.

## Data Availability

Data sharing is not applicable to this article as no datasets were generated or analyzed during the current study.
